# MicroRNAs and pro-inflammatory cytokines as candidate biomarkers for recent-onset psychosis

**DOI:** 10.1186/s12888-023-05136-6

**Published:** 2023-08-29

**Authors:** Ali Reza Shafiee-Kandjani, Negin Nezhadettehad, Sara Farhang, Richard Bruggeman, Dariush Shanebandi, Mohammadbagher Hassanzadeh, Hosein Azizi

**Affiliations:** 1 Faculty of Medicine, Tabriz University of Medical Sciences, Tabriz, Iran; 2 University Medical Center Groningen, Groningen, Netherlands; 3 Research Center of Psychiatry and Behavioral Sciences, Tabriz University of Medical Sciences, Tabriz, Iran; 4 Immunology Research Center, Tabriz University of Medical Sciences, Tabriz, Iran; 5 Women’s Reproductive Health Research Center, Tabriz University of Medical Sciences, Tabriz, Iran

**Keywords:** Schizophrenia, miRNAs, Psychoneuroimmunology, Tumor necrosis factor-alpha, Interleukin-1beta: Interleukin-6

## Abstract

**Background:**

Recent studies on the schizophrenia spectrum and other psychotic disorders showed that alternation of immune system components, particularly microRNAs (miRNAs) and pro-inflammatory compounds, plays a significant role in developing the illness. The study aimed to evaluate serum expression of the miRNA-26a, miRNA-106a, and miRNA-125b as genetic factors and serum levels of IL-6, IL-1β, and TNF-α as pro-inflammatory factors in an IranianAzeri population.

**Methods:**

Forty patients with recent-onset non-affective psychosis and 40 healthy people as a control group were involved. Expression levels of miRNAs and serum levels of the cytokines were measured using RT-qPCR and ELISA, respectively. T-test, receiver operating characteristics (ROC), and spearman correlation coefficient were carried out data analysis.

**Results:**

Findings showed higher levels of IL-6, IL-1β, TNF-α, miR-26a, and miR-106a in the plasma of the patients’ group compared with the control. miRNA-26a showed a statistically significant higher level (*p* < .003) compared to the control group, with AUC = 0.84 (95% CI: 0.77 to 0.93, *P* < .001) and cut-off point = 0.17 in comparison to other miRNAs as mentioned above; in this regard, it might be a suggestive biomarker for schizophrenia in the early stage of the illness. Moreover, miRNAs’ expression level was not substantially associated with the level of any measured cytokines above.

**Conclusions:**

miR-26a might be a suggestive biomarker for schizophrenia in the early stage of the illness. Given that the relationship between other miRNAs and cytokines is not yet well understood; accordingly, there are encouragement and support for continued research in this fascinating field.

## Background

Recent studies show that the immune system and its components have a crucial role in mental disorders, particularly schizophrenia [1]. Inappropriate immunological responses can impact the brain of the patients(Lesh et al., 2018), who have different levels of pro-inflammatory cytokines, notably interleukin-6 (IL-6), interleukin-1beta (IL-1β), and Tumor Necrosis Factor-alpha (TNF-α) in their peripheral blood or cerebrospinal fluid [2]. IL-6 is the most well-known cytokine related to schizophrenia [1]. Immunological responses and cytokines affect the neural system functioning and play an essential role in developing mental disorders [3]. Hypothetically, adjustments in cytokines’ level can solely result from psychological tension or sleep deficiency linked to the primary stages of the diseases [4]. On the other hand, they can alter neurotransmitters’ metabolism and neuroendocrine hormones, consequently affect neural expansion and neurodegeneration [5].

Immune cells mainly produced TNF-α, which has a remarkable role in the pathogenesis of schizophrenia by affecting the dendritic development of cortical neurons [6]. A neuropathological study indicated high transcription levels of pro-inflammatory cytokines, especially in the subgenual cingulate white matter; this was also replicated in the first-episode psychosis [7]. An increased level of IL-1β, as a pro-inflammatory cytokine, can lead to inflammation in the first episode of schizophrenia [6]; therefore, the evidence provides new information on monocytes’ over-activation in schizophrenia [8]. Latest investigations reported high levels of TNF-α and IL-1β in patients with the first episode of psychosis and those taking antipsychotic agents [9]; however, IL1-β levels decreased after remission [10]. Studies showed that serum levels of TNF-α and IL-1β were high in chronic patients receiving antipsychotics; nevertheless, they were low in patients with the first-episode of psychosis [11].

Based on several studies, a particular type of non-coding Ribonucleic Acid (RNA) -known as microRNAs- may participate in the pathogenesis of schizophrenia [12]. MicroRNAs are single-stranded molecules containing 21–23 nucleotides that are essential factors in regulating gene expression [13]. It is supposed that miRNAs directly target about one-third of human genes, and consequently, they reduce their target gene expression by being bound to the 3’UTR region of mRNAs [14]. A very feeble connection between miRNAs and the development of the neural system has been recently identified [15]. Also, recognizing changes in prenatal gene expression networks is a preferred approach to study the determinants involved in schizophrenia, and profiling miRNAs’ expression is the focal point of recent studies [16]. According to new studies, miR-26a, miR-106a, and miR-125b adjust the expression level of genes involved in the immune response [17] (Viswambharan et al., 2017), although the leading pathogenesis is unclear in schizophrenia [16, 18].

To the best of our knowledge, in the Iranian-Azeri ethnicity, the possible role of miRNAs and pro-inflammatory cytokines in recent-onset psychosis has not already been studied. Moreover, due to the possible advantages of these factors on the new psychopharmacological approaches; we aimed to examine the plasma expression of three miRNAs (106a, 26a, 125b) and plasma level of three pro-inflammatory cytokines (TNF-α, IL-6, and IL-1β) and explore the correlation between these factors among patients with schizophrenia spectrum disorders.

## Methods

### Study design and setting

This study was conducted within the outline of The Azeri Recent Onset Acute Phase.

Psychosis Survey (ARAS Cohort) Study Protocol [19]. All applicants and their legal guardians gave written informed consent based on the Helsinki Declaration, a statement of ethical values for medical research concerning human samples, including research involving recognizable human material and data [20].

### Participants

A sample of 40 people was taken after applying the inclusion and exclusion criteria using the convenience sampling method [1]. Simultaneously, the control group was matched with 40 counterparts regarding age, gender, and education level.

Adult subjects were patients with schizophrenia spectrum disorders aged 18–45 who had a recent-onset (less than two years) of non-affective psychosis. The study involved schizophrenia spectrum disorders including schizophreniform disorder, delusional disorder, brief psychotic disorder, schizotypal personality disorder, and schizoaffective disorder based on the Diagnostic and Statistical Manual of Mental Disorders 5th edition (DSM-5) [21]. Thus, cases with a psychotic disorder due to a general medical condition, substance-induced psychotic disorder, dissociative disorder, or affective psychosis were excluded. A local statement carefully chose the control group at the same health care facilities; furthermore, lack of current or past psychiatric conditions was confirmed by Structured Clinical Interview for Axis I Disorders (SCID-I) [22]; also, those with a verified history of psychiatric disorder in their first-degree relatives were excluded too.

### Measures

Symptoms of intensity were evaluated using the Positive and Negative Syndrome Scale (PANSS). In this questionnaire, high scores are indicative of a severe condition. The reliability and Cronbach’s alpha of this scale were 0.73, 0.83, and 0.79 for the subscales of positive symptoms, negative symptoms, and general disorder, respectively [23]. The SCID-1 questions were generally understandable and acceptable for Iranian people. The Persian translation of the SCID-1 yields diagnoses with adequate reliability and validity in an Iranian clinical population, helping the cross-cultural use of instrument [24]. The Global Assessment of Functioning (GAF) is measured between the case and control group [25].

### Procedure

The peripheral blood sample was collected by sterile venipuncture into tubes with an interior coated spray-dried K2EDTA (Dipotassium Ethylene Diamine Tetra Acetic Acid) [26]. This sample was centrifuged at 23ºC for 10 min at 1300 Revolutions Per Minute (RPM). After centrifugation and plasma separation, samples were split to determine the serum levels of cytokines and plasma expression of miRNAs by Enzyme-linked Immunosorbent Assay (ELISA) and Real-Time Quantitative Polymerase Chain Reaction (RT-qPCR), respectively. Samples were kept at -80 °C after segregating and centrifugation until the analyses were conducted.

Whole RNA was extracted from 250 µl of plasma utilizing TRIzol [27] reagent according to the manufacturer’s recommendations. The extracted RNAs were assessed by 2000 C spectrophotometer [28]. One hundred nanograms of the obtained RNA were treated with DNase I. Stem-loop primers specific for each miRNA, and a complementary DNA (cDNA) reverse transcription kit was used for cDNA synthesis [29].

The SYBR Green Master Mix was applied to quantify the expression levels following the manufacturer’s protocol. Real-time PCR was done using a standard SYBR Green PCR master mix protocol on a Light-Cycler 96 system according to the instructions: 95 ° C for 3 minutes, followed by 45 circles at 95 ° C for 10 secs and 60 ° C for 60 secs. Cycle threshold rates were used for relative quantification by using the 2 (-Delta Delta C(T)) method introduced by Livak [30], and all values were standardized to the *U6* housekeeping gene. Primer and stem-loop sequences are outlined in Table 1. A sandwich ELISA kit measured plasma levels of IL-6, IL-1β, and TNF-α.

**Table 1:** RT-qPCR^a^ miRNAs primers

Primer Name	Forward	Reverse
miRNA-125b	TGACCGTAGTCCCTGAGACCCC	CCAGTGCAGGGTCCGAGGTA
miRNA-106a	GGGCCGGGAAAAGTGCTTACC	CCAGTGCAGGGTCCGAGGTA
miRNA-26a	GGGCCGGGGTTCAAGTAATC	CCAGTGCAGGGTCCGAGGTA
Stem-loop U6	GTCGTATCCAGTGCAGGGTCCGAGGTATTCGCACTGGATACGACAAAAATAT	
Stem-loop-125b	GTCGTATCCAGTGCAGGGTCCGAGGTATTCGCACTGGATACGACTCACAA	
Stem-loop-106a	GTCGTATCCAGTGCAGGGTCCGAGGTATTCGCACTGGATACGACCTACCT	
Stem-loop-26a	GTCGTATCCAGTGCAGGGTCCGAGGTATTCGCACTGGATACGACGCCTAT	

^a^ Real-time quantitative poly chain reaction

### Statistical analysis

For statistical analysis, GraphPad Prism (version 6.0 for Windows) was utilized. The outcome confirmed the assumption that the two specimens originate from different populations. Data are presented as the mean ± standard error or standard deviation. Two tests, Shapiro-Wilk and Kolmogorov-Smirnov, were used to check the normal distribution of data. Due to the uncertainty of the general population’s standard deviation, a t-test was used to model the data and draw conclusions. Mann–Whitney U test was used when data distribution was not normal. A *P*-value < 0.05 was considered significant. The diagnostic value of diverse variables between the two groups (as a binary classifier) was examined by a receiver operating characteristics (ROC) curve. For this test, the area under the ROC curve (AUC) reflected the mark variables predictive value to trustworthy distinguish among patients and healthy controls. AUC of 0.50 indicates no diagnostic value, while AUC more than 0.50 designates a higher probability of success and lower randomness of the results. The correlation between all the above miRNAs’ expression and miRNA-26a’s expression with cytokines levels (IL-6, IL-1β, and TNF-α) was investigated using the Spearman correlation coefficient.

### Ethical considerations

In this study, participation was entirely voluntary, patient information remained confidential, the patients were under routine treatments and free to leave at any stage of the study.

## Results

We analyzed 40 patients with schizophrenia spectrum disorders and 40 healthy controls with Azeri ethnic background. The mean age of patients was 29.8 ± 9.6, and the control group was 30.3 ± 8.6. Out of 67.5% of patients and 57.4% of the control group were males (Table 2).

**Table 2:** Characteristics of the study participants ^a^

	schizophrenia spectrum	Healthy control	*P*-value
n*	40	40	
Male to Female Ratio	2.0	1.3	0.356
Mean age (SD)	29.8(9.6)	30.3 (8.6)	0.788
Married^b^	22.5	45	0.009**
Employment	14 (35.0)	31 (77.5)	< 0.001**
Mean years of education (SD)	9.1(3.2)	15.6(2.5)	0.045**
The score of positive symptoms	16.7(4.3)	-	-
The score of negative symptoms	10.0(3.3)	-	-
Global assessment of functioning score	30.1 (6.4)	91.1(5.1)	0.001**

^a^ Values are mean ± S.D.

^b^ Values are %

* n = number of cases where required data were available, ** statistically significant *P*-value

Compared with the controls, as shown in Table 3, patients with the first-onset psychosis of schizophrenia spectrum disorders showed statistically significant upregulation of miR-26a (*P* < .003) and miR-106a (*P* = .005). On the other hand, the expression of miR-125b in the case group was lower (*P* = .480) than healthy controls, but this downregulation was not statistically significant.

**Table 3:** Comparison of miRNAs and cytokines^a^ in the study groups

Cytokines/Primers	Cases	Control	*P*-value
IL-6	43.20 ± 3.87	7.010 ± 1.45	< 0.001 *
IL-1β	164.125 ± 41.964	125.225 ± 25.596	< 0.001*
TNF-α	43.907 ± 29.873	24.4655 ± 10.631	< 0.002*
miRNA-26a	1.424 ± 0.319	0.202 ± 0.034	< 0.003*
miRNA-106a	0.176 ± 0.038	0.033 ± 0.007	< 0.005*
miRNA-125b	2.313 ± 4.276	3.096 ± 5.533	< 0.480

* statistically significant *P*-value

There were higher levels of IL-6 (*P* < .001), IL-1β (*P* < .001), and TNF-α (*P* < .002) in the case group in comparison with the control group.

**Fig. 1:**
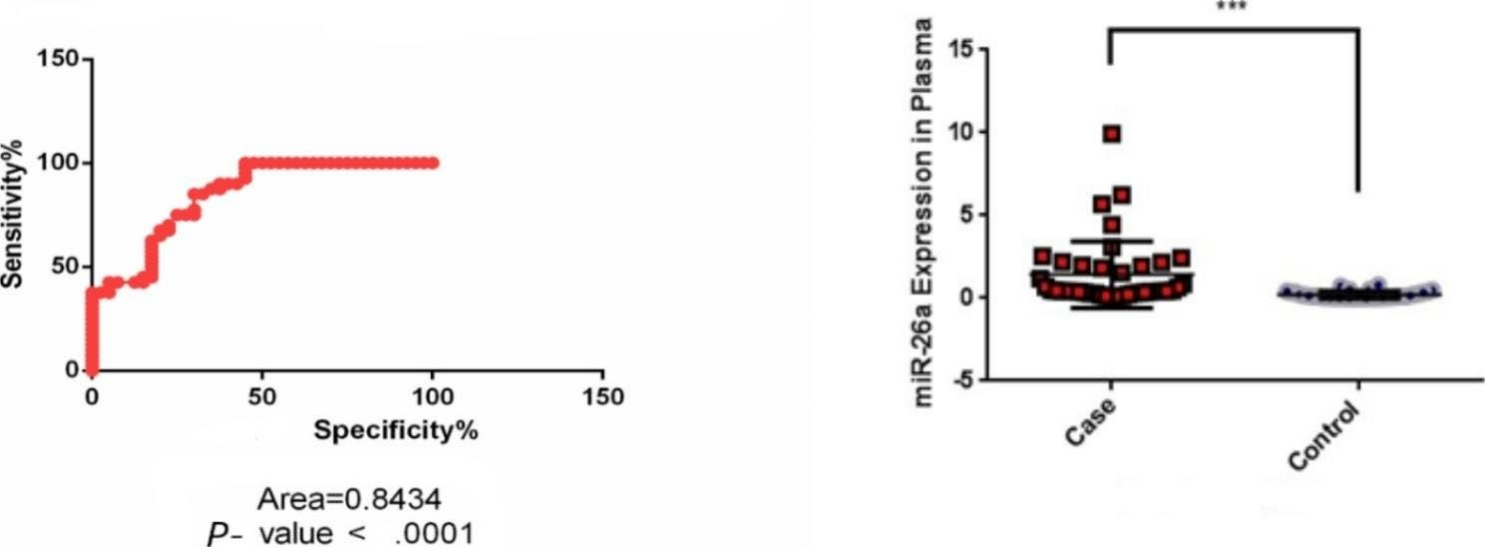
ROC of miR-26a gene expression in plasma (Left), the Plasma expression of miR-26a (Right). ROC of miRNA-26a with AUC = 0.8434 (*P-*value < 0.0001) Cut-off point 0.1726 demonstrated promising values. MiR-26a expression in plasma compared with the control group showed a statistically significant difference (*P-*value = .0003)

The AUC was carried out for the expression level of miR-26a, miR-106a, and miR-125b to understand their value as a potential biomarker for recent-onset psychosis. Between miRNAs, the miR-26a with AUC = 0.843 (95% CI: 0.769 to 0.937, *P* < .0001) and cut-off point of 0.172, demonstrated a promising values as shown in Fig. 1, however regarding our findings, miR-125b with AUC = 0.625 (95% CI: 0.373 to 0.661, *P* = .0525) and miR-106a with AUC = 0.548 (95% CI: 0.403 to 0.693, *P* = .461) were not capable biomarker.

We also evaluated the plasma level of the TNF-α, IL-1β, and IL-6; consequently, all of these cytokines revealed trustable AUC levels to be a possible diagnostic biomarker. AUC for the plasma concentration of the TNF-α was 0.680 (95% CI: 0.558 to 0.802, *P* = .005) with a cut-off point of 24.47. AUC for the plasma concentration of the IL-1β was 0.792 (95% CI: 0.693 to 0.891, *P* < .0001) with a cut-off point of 125.17. In IL-6, AUC was 0.9584 (95% CI: 0.918–0.998, *P* < .0001) with a cut-off point of 9.32.

**Fig. 2:**
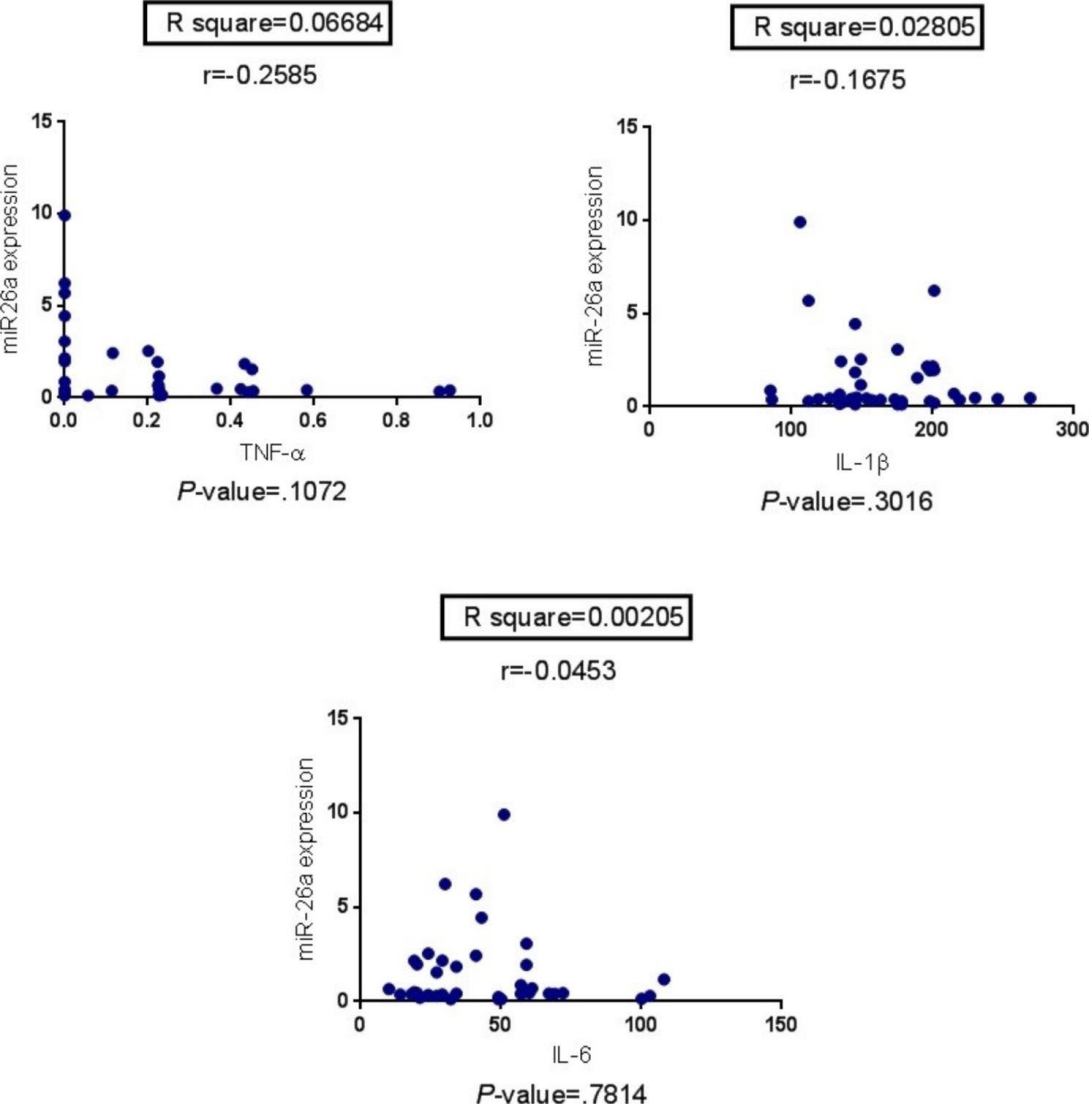
Correlation of miRNA-26a with cytokines^a^. ^a^ There was not significantly correlated with any cytokines

According to the Spearman correlation coefficient and the R-squares, there was no significant relationship between any miRNAs pair (*P* > .05). As shown in Fig. 2, the miR-26a’s level was not significantly correlated with any measured cytokines.

## Discussion

The heritability of schizophrenia is estimated to be about 80%; however, most established robust signals lacked clear biological significance, indicating that most attempts to identify a genetic cause for the disease have not been as helpful as predicted. Nevertheless, mounting evidence appears to implicate hereditary dysregulation, possibly triggered by the degradation of miRNAs’ regulating functions, in the pathophysiology of schizophrenia. In the present study, miR-26a and miR-106a expression levels showed an upregulation, in which the former was much more upregulated than the latter; meanwhile, miR-125b revealed significant downregulation compared to the control group regarding differences in the predictive value, sensitivity, and specificity of these factors.

It has recently been found that miR-26b was expressed at lower levels in the prefrontal postmortem cortex tissue of the patient with schizophrenia than control subject s [31]. The results of the mentioned study were inconsistent with our study results. This disagreement can be related to the fact that miR-26a and − 26b are miRNAs families showing a highly unspoiled sequence and sharing the same seed sequence [32]. Worth mentioning, miR-26s are differentially expressed in tissues and are highly expressed in the brain [33]. These observations were taken together with the expression data available in public databases, leading to hypothesizing that widely divergent or conflicting results, in large part due to the variations in sample status and the tissues used. It is impossible to say if changes in miRNA in postmortem brain tissue are causative, stochastic, or the result of long-term therapy and physical conditions. Another reason for this difference could be explained by suggesting that age significantly impacts the complex mechanism of miRNA expression in the brain; in this regard, a dynamic change in miRNA expression has been found during brain maturation [34].

In another recent study in Turkey, between 16 patients with schizophrenia and 16 healthy controls, all patients with schizophrenia showed statistically significant upregulation in following miRNAs levels in comparison to the control group: miR106b-5p (*P*-value = 0.002), miR125a-3p (*P*-value < 0.001), and miR-125b-3p (*P*-value = 0.018). This study’s findings showed a higher level of the miR-125b in contradiction to our study results, which was showed a lower level of the miR-125b. Besides, in this study, the miR-106a level was higher than the control group with an insignificant P = .089, which is inconsistent with the current study’s findings (*P*-value = 0.0005). Regarding that there are differences in the type of studied tissues, the method of conducting and designing with the present study, such a discrepancy would be expected; however, due to the possible role of these factors in coding and expressing microRNAs, further studies on tissue other than peripheral blood, such as brain tissue with sufficient sample size, may help clarify this topic.

The role of miR-125b was investigated in individuals with a history of childhood trauma in the brain tissue of humans and mice stressed in vivo [18]. The miR-125b is analyzed in different samples (blood of adult subjects exposed to childhood trauma, the brain of rats exposed to prenatal stress, and human hippocampal progenitor cells treated with cortisol), and they identified miR-125b as a down-regulated miRNA in all three datasets. Some studies found that miR-125b could be involved in the mechanism associated with long-term stress and how it leads to progression to schizophrenia in the future. The results of the mentioned studies were consistent with the present study’s findings, indicating a reduced level of miR-125b and confirm the possibility of the crucial role of this factor in schizophrenia.

There were several investigations and recent developments on miRNA and connections with schizophrenia disorder. Zhang et al. [35] investigated that miRNAs have come to light as possible participants in the pathophysiology of schizophrenia by having an impact on these systems. These miRNAs regulate the stability and translation of hundreds of target transcripts, which has an impact on the entire gene network. There may be improved approaches in the case management of schizophrenia patients to diagnose and treatment if it is understood how these variations in miRNAs change the critical related signaling pathways that drive the development and progression of the disease [36]. Besides, previous findings provided the accumulated evidence connecting miRNA dysregulation and dysfunction with schizophrenia, other psychiatric disorders and the potential of miRNAs as biomarkers or therapeutics for these disorders [37, 38].

Considering the possible role of the miR-125b in patients with a history of childhood trauma could allow the early detection of vulnerable subjects for schizophrenia. It could provide the basis for the development of preventive, therapeutic strategies. The level of miR-26a in the present study had a significant upregulation compared to the control group; therefore, according to the calculated AUC for the expression level of miR-26a, some aspects of our results may have translational importance because the multiple linear regression models indicated an association of psychosis status with the changes observed in miR-26a expression and patients’ global functioning. MicroRNA-26b had a higher predictive value than the other two types (miR-106a and miR-125b); although longer longitudinal studies should be conducted, this is, to our knowledge, the first time that the miR-26b in the peripheral blood sample of patients with recent-onset psychosis is described as a possible biomarker for early detection of the disorder. Notwithstanding, few studies have been done in this regard. Some studies showed altered expression levels of miR-26a in patients with psychiatric disorders probably related to the role of miR-26a in prose-related plasma levels of neurotrophins (brain-derived neurotrophic factor [BDNF) production. The possible changes in BDNF have been proven [39] (Martinez-Cengotitabengoa et al., 2016), so it would be crucial to study these variants’ involvement in psychiatric disorders pathogenesis in large case-control samples.

To clarify miR-26a, 106a, and − 125b’s possible role in directing pro-inflammatory cytokines, the relationship between these microRNAs expressions was compared with three pro-inflammatory levels factors (TNF-α, IL-6, and IL-1β). Although such association was proposed in previous studies on different tissues and disease, such a relationship was rejected in the present study. For example, Chen et al. showed the entire IL-6 transcript, not have a suitable sequence for addressing interaction with miR-26 as a gene silencing function [40].

The present study also established that plasma levels of the three inflammatory factors IL-6, IL-1β, and TNF-α in participants with recent-onset psychosis were higher than the healthy control group, so these three cytokine levels might help diagnose the schizophrenia disorder alongside the traditional diagnostic psychiatry interview. In a 2015 study on 30 patients with schizophrenia, IL-6 and TNF-α levels were significantly elevated in the patient group treated with antipsychotics; this study also showed an immunological response in schizophrenia, despite the lack of factors that cause inflammation [41].

An updated review in 2019 reported increasing levels of IL-6, TNF-α, and IL-1β in different groups of patients, including first-time psychosis without medication and patients with first-time psychosis who were often treated with antipsychotics and chronic patients and patients with acute recurrence [42]. This study showed a possible contributing role for cytokines in schizophrenia pathogenesis.

The clinical condition can also alter cytokine changes in schizophrenia. IL-6 has been suggested as a state-related marker because it has increased during the acute phase and recent relapse-with previous antipsychotic use- and in patients with the first episode of psychosis without medication and has been normalized with long-term antipsychotic treatment. Our recent study results were in line with these findings, even though we did not chronologically measure these cytokine levels to determine their changes in the course of the illness. According to the studies, antipsychotics affect IL-6 levels; however, it does not return to normal levels, indicating the active macrophage system in schizophrenia [43].

The TNF-α investigation results in all cases with and without medication showed an increased level of this cytokine. As in the meta-analysis [1], the level of cytokines in patients with psychosis for the first time without treatment as well as follow-up and re-checking after treatment has been shown an increased level of this factor in the baseline and even after treatment. Therefore, this could be proof of the trait-related nature of this factor [44]. The level of TNF-α in patients with a chronic course has also been increased, which might be a hint for TNF-α ability related to schizophrenia’s course [9].

## Conclusions

Recent studies showed schizophrenia associated with several abnormalities in the miRNAs and cytokines. Alterations in cytokine levels and miRNAs expression may precede the first-episode of psychosis; thus, these serum markers might be useful for early illness detection strategies. The current study’s results showed that the miR-26a could be suggested as a potential biomarker for schizophrenia and related disorder diagnosis in the early stages. More analysis needs to be undertaken to clarify the miRNAs and the immune system’s involvement in this regard.

It is recommended that miRNA levels assess by disease severity and clinical profiles to provide reliable evidence for the concoctions between higher levels of IL-6, IL-1β, TNF-α, and mi-RNAs in patients with schizophrenia in comparison with controls. This is a departure point for conducting future studies.

## Data Availability

The datasets generated and/or analysed during the current study are available from the corresponding author on reasonable request.
